# Persistent bacteremia caused by *Ralstonia pickettii* and *Microbacterium*: a case report

**DOI:** 10.1186/s12879-024-09228-w

**Published:** 2024-03-18

**Authors:** Jinwen Wang, Yu Song, Siqin Liu, Xudong Jang, Lina Zhang

**Affiliations:** Department of Laboratory Medicine, Daqing Oilfield General Hospital, Daqing, China

**Keywords:** *Ralstonia pickettii*, *Microbacterium*, Bacteremia, Rapid antimicrobial susceptibility testing, Septic shock

## Abstract

**Background:**

*Ralstonia pickettii* is a low virulent, gram-negative bacillus that is rarely associated with human infections and may cause bacteremia. *Microbacterium* species are gram-positive coryneforms that are generally considered as a contaminant in Gram staining of blood cultures, especially when the time to positivity is longer than 48 h. Both these bacterial species are emerging opportunistic pathogens that may occasionally cause serious infections and even life-threatening health conditions.

**Case presentation:**

Here, we report the case of a patient with bacteremia caused by both R. *pickettii* and *Microbacterium.* We advocate for providers to order rapid antibiotic susceptibility testing, since our patient’s suffered two kinds of rare pathogens with the opposite of drug sensitivity results to imipenem.

**Conclusions:**

Our case present a patient suffered septic shock caused by *R. pickettii* and *Microbacterium*. Improving the antibiotic management based on the result of antimicrobial susceptibility tests is the key of successful treatment.

## Background

Nonfermentative gram-negative bacilli are mostly opportunistic pathogens, and they are not only widely distributed in nature but also occur in hospital settings, leading to hospital-acquired infections. *Ralstonia* is a new genus established in 1995. It was formerly designated as *Burkholderia*. *R. pickettii* was first isolated in 1973 and named as *Pseudomonas pickettii* or *Burkholderia pickettii*. *Ralstonia* spp. are rarely isolated from clinical specimens, and the most commonly isolated strain from clinical specimens is *R. pickettii* [1]. *R*. *pickettii* infections have been previously reported in the literature. This indicates that the organism might be a more widespread pathogen than previously thought. Although it is of low virulence, it has been associated with varying severity such as sepsis, chest infections, pneumonia, bone and joint infections, and peritonitis [2–4]as well as their resistance to many classes of antibiotics. As an emerging opportunistic pathogen associated with several bacteremia outbreaks reported in recent years, it is responsible for rare nosocomial infections in immunocompromised and multimorbid individuals. Isolates reported in papers showed different resistance profiles, especially in β-lactam antibiotics which should be the key of the therapeutic management.

The genus *Microbacterium* was first proposed by Orla Jensen in 1919. Since then, its classification underwent several changes. In 1983, Collins et al. redefined *Microbacterium* [5]. At present, the genus *Microbacterium* includes 156 species (https://lpsn.dsmz.de/search?word=Microbacterium). The strain was not isolated from clinical samples until the 1990s [6]. The most common isolates confirmed in clinical samples were M. *oxydans*, *M. paraoxydans*, and *M. foliorum* [7]. There are few reports on *Microbacterium* isolated from clinical samples and the resistance profiles are quite different especially the resistance rate to vancomycin and meropenem [7–9]. The gradually increased amount of investigation report supports the genus *Microbacterium* to be opportunistic human pathogens. Major of them were catheter-related infection with bacteremia or peritonitis [10]. Despite all this, the epidemiology and pathogenic potential of it are yet to be investigated more profoundly.

## Case presentation

**Fig. 1:**
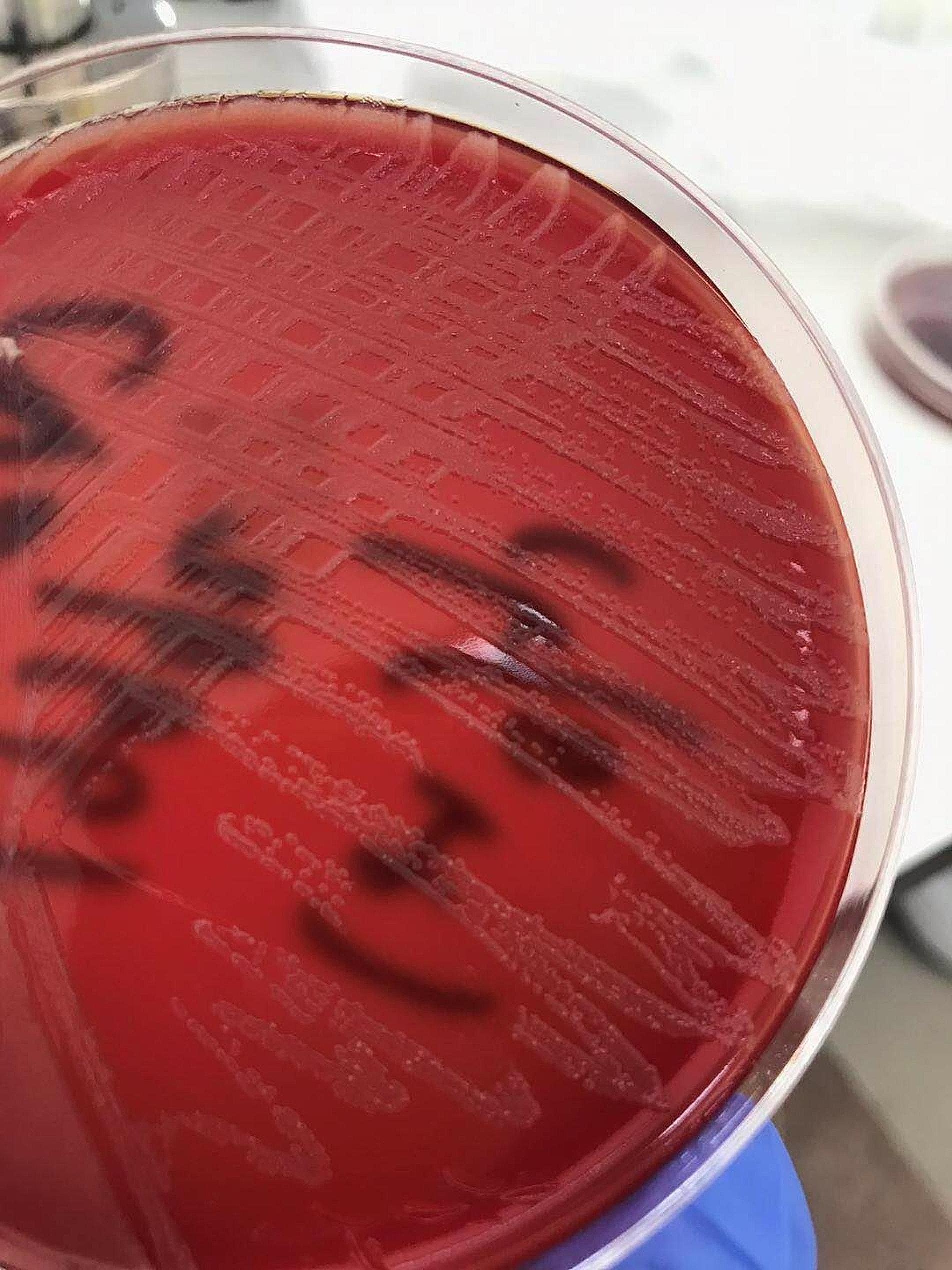
Fewer white colonies on the blood culture plate of the positive blood culture bottle along with the colonies of *R. pickettii* were seen

**Fig. 2:**
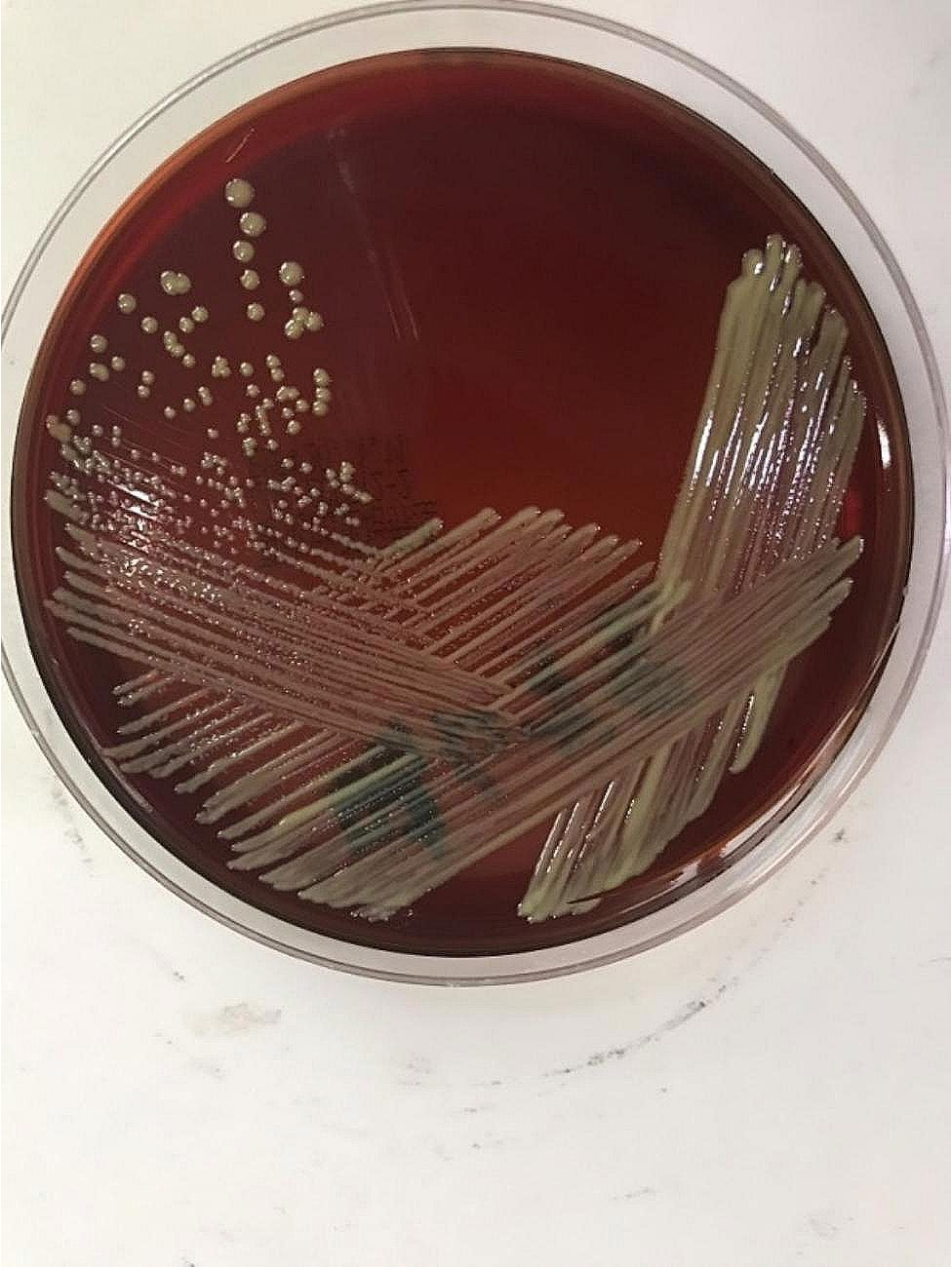
After 48 h of purification and incubation, the colonies became yellow

**Fig. 3:**
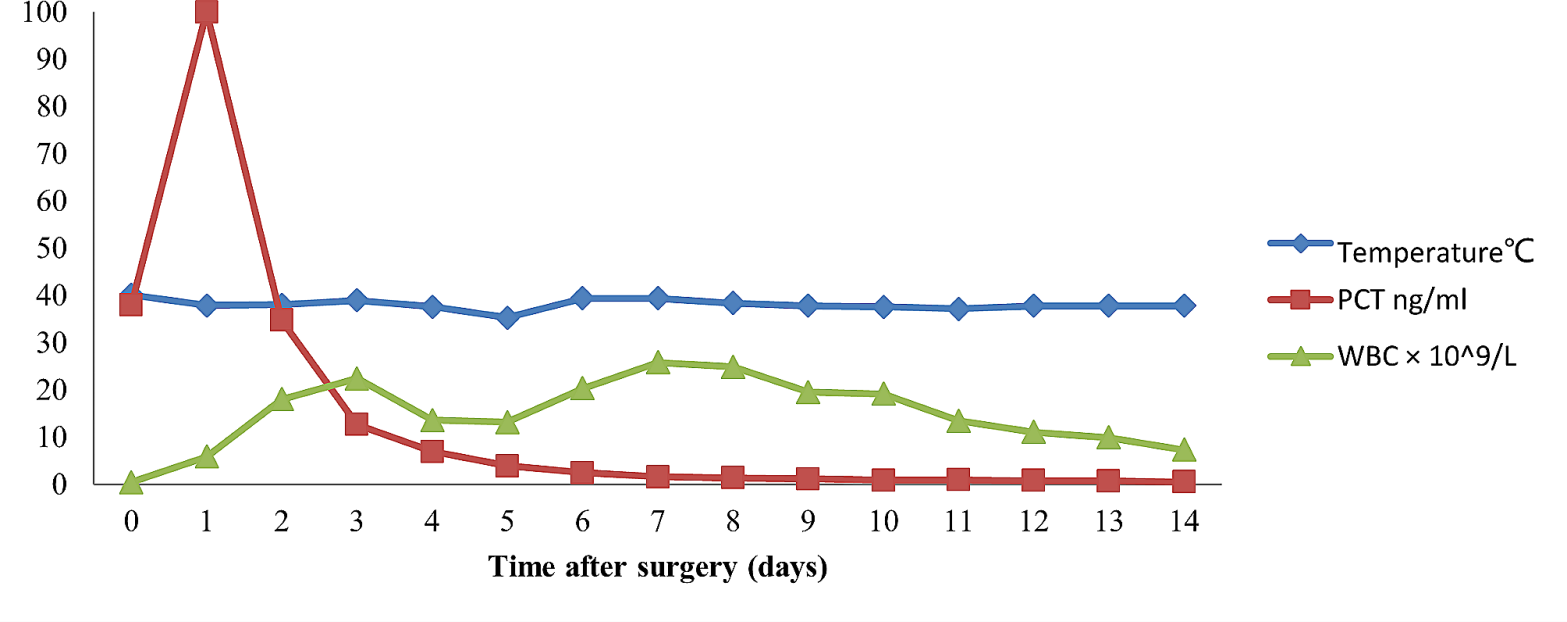
The change of infection index after surgery during treatment

In 2020, a 50-year-old female was admitted to our hospital because of pain due to a popliteal cyst on her left knee, which was detected 6 years ago. She has no notable pathological history other than Hashimoto’s thyroiditis, with the treatment of consists in daily assumption of synthetic levothyroxine. The level of anti-thyroglobulin antibodies (ATG) was 347.6IU/mL. Physical examination did not reveal any apparent focus of infection before surgery. The total leucocyte and neutrophil counts were 4.2 × 10^9^/L and 1.84 × 10^9^/L, respectively, and the platelet count was 203 × 10^9^/L. Two hours after receiving resection for the popliteal cyst, she developed a fever of 41 °C with chills and skin flushing throughout the body. Her blood pressure was 88/47 mmHg; the total leucocyte and neutrophil counts were 0.49 × 10^9^/L and 0.24 × 10^9^/L, respectively, and the platelet count was 130 × 10^9^/L. Procalcitonin level was 30 ng/mL. On the basis of the symptoms, the patient was considered to have septic shock, and the Acute Physiology and Chronic Health Evaluation (APACHE) II score was 20.She was transferred to the intensive care unit immediately, received a fluid replacement and was administered epinephrine and dopamine through a micropump. Imipenem (500 mg) was infused intravenously every 6 h, following which the total leukocyte count increased to 18 × 10^9^/L. Two sample bottles of blood collected 2 h after the operation were cultured aerobically, and the rapid antimicrobial susceptibility test was performed as per Clinical and Laboratory Standards Institute (CLSI)guidelines by Kirby-Bauer disk diffusion method on Muller Hilton agar, on the basis of a positive test result after 28 h of incubation. The bacterial pathogen was identified as *Ralstonia pickettii* by the Vitek 2 Gram-negative identification card(GN), According to the results of the antimicrobial susceptibility test provided by the Vitek 2 AST-GN335 card, the antibacterial therapy was changed to cefoperazone-sulbactam (3 g every 8 h). Following the treatment, the body temperature initially decreased to 37 °C and later again increased to 39 °C during 2 days after surgery. The leukocyte count also initially decreased and then gradually increased from 13.2 × 10^9^/L to 25.8 × 10^9^/L. Procalcitonin level was 12.07 ng/mL. The disease progressed rapidly, and multiple organ dysfunction syndrome (mods), renal insufficiency, liver dysfunction followed. Hemodialysis was performed. The blood pressure dropped to 40/21 mmHg, and electric defibrillation was given due to the frequent ventricular tachycardia. Subsequently, we found the growth of other fewer white colonies on the culture plate of the positive blood culture bottle along with the colonies of *R. pickettii* (Fig. 1), which showed yellow after purification and incubation in blood agar plates after 48 h (Fig. 2). The organism appeared as a gram-positive coryneform on smear; this finding was consistent with the colonies obtained from the other three positive blood cultures in succession. These blood cultures were performed 2 days after surgery and included 2 bottles cultured aerobically and 1 bottle cultured anaerobically, which showed bacterial growth at 62 h, 55 h, and 97 h, respectively. Bacterial DNA extraction, PCR amplification and DNA sequencing of the 16 S rRNA genes were performed. The sequences of the PCR products were compared with known 16 S rRNA gene sequences in the GenBank database. The analysis revealed 99.7% similarity between the isolate and *Microbacterium laevaniformans*(GenBank sequence ID:NZ SKYO000021.1). The patient was placed on a combination treatment of intravenous cefoperazone-sulbactam (3 g every 8 h) and levofloxacin (500 mg once daily) immediately after the identification of *Microbacterium* in blood culture. The patient remained stable after completing her 2-week course of antibiotics and was transferred to the general ward, with ATG level reduced to 211 IU/mL 13days later. The changes of infection index during the treatment of septic shock were shown in Fig. 3 and Table 1.

**Table 1:** Antibiotic management and clinical outcomes of the patient

Time after surgery(days)	Temperature (℃)	WBC×10^9^/L	PLT×10^9^/L	ALT (U/L)	AST (U/L)	Tbil (umol/L)	ALB (g/L)	CREA (umol/L)	PCT (ng/ml)	Antibiotic treatment
0	40	0.49	130	/	/	/	/	/	38	IPM
1	37.9	1.3	120	57.3	132.4	28.6	27.2	116.5	100	IPM
2	38	18.1	52	41.9	75.4	26.5	35.1	84	34.78	IPM
3	39	22.4	22	68.3	159.6	54.2	23.1	78	12.07	IPM
4	37.6	13.6	21	328.1	421.6	86.9	24	32	6.98	SCF
5	37	20.2	39	336.9	369.4	114.1	27.9	39	3.95	SCF
7	39.4	25.8	48	4.9	123.9	153.4	28.3	34	1.6	SCF + LEV
9	37.8	22.6	88	36.0	49.4	103.9	22.8	33	1.17	SCF + LEV
14	37.8	8.6	288	55.2	72.2	57.1	31.3	33	0.571	SCF + LEV

*Note*: WBC: white blood cell counts, PLT: platelet counts, ALT: alanine aminotransferase, AST: aspartate aminotransferase, Tbil: total bilirubin, ALB: albumin, CREA: creatinine, PCT: procalcitonin, IPM: Imipenem, SCF: Cefoperazone-sulbactam, LEV:Levofloxacin

## Discussion and conclusions

In recent years, *R. pickettii* has been reportedly isolated from various samples, including blood, urine, and cerebrospinal fluid. These organisms are prevalent in several types of water supply, including municipal drinking water, bottled water, dental water supply, hospital water supply, space shuttle water system, standard purified water, laboratory-based high-purity water system, and industrial ultrapure/high-purity water supply [11]. *R. pickettii* has also been detected in biofilms on plastic industrial water pipelines, and it has been shown to cause infections, occasionally serious, such as bacteremia, pneumonia, endocarditis, primary peritonitis, and venous catheter-related infection [12].

In this article, the antimicrobial susceptibility of the isolated *R. pickettii* strain was determined by the broth microdilution method using the VITEK 2™ system. As shown in Table 2, the strain was resistant to amikacin, aztreonam, minocycline, piperacillin/tazobactam, and tobramycin; intermediate to cephalosporins; and susceptible to trimethoprim/sulfamethoxazole, ciprofloxacin, tigecycline, cefoperazone-sulbactam, imipenem, and levofloxacin. These results were similar to those reported by Monica Basso et al [4]. The strain reported by Sheng Chaojun et al. was susceptible to cephalosporins [13]. Our isolated strain was resistant to tobramycin; this finding was consistent with the results of Yan Tao et al [1], but inconsistent with those reported by Monica Basso et al [4]. Moreover, our isolated strain was resistant to meropenem; this observation differed from the results reported in the literature, except for the study of Monica Basso et al. Differences were also found in sensitivity to other antibiotics, particularly carbapenems and aminoglycosides, which might be due to the presence of two inducible β-lactamases, namely blaOXA-22 and blaOXA-60 [14]. The existence of these beta lactamases require further studies to verify in the isolated strain. However, several studies have reported good results for *R. pickettii* infections treated with piperacillin/tazobactam, meropenem, ciprofloxacin, amikacin, or the combination of cephalosporins and aminoglycosides [15].

**Table 2:** MIC values for *Ralstonia pickettii* clinical isolates described in this study

Antibiotics	Antibiotic MIC values (mg/L)^a^
Amikacin	> 64 (R)
Aztreonam	> 64 (R)
Trimethoprim/sulfamethoxazole	40 (S)
Ciprofloxacin	< 0.25 (S)
Meropenem	8 (R)
Minocycline	≥ 16 (R)
Piperacillin/tazobactam	≥ 128 (R)
Tegacyclin	1 (S)
Cefepime	16 (I)
Cefoperazone-Sulbactam	≤ 8 (S)
Ceftazidime	16 (I)
Tobramycin	≥ 16 (R)
Imipenem	2 (S)
Levofloxacin	0.5 (S)

*Note*: ^a^MIC values were interpreted according to Clinical and Laboratory Standards Institute (CLSI) criteria for *Pseudomonas aeruginosa*

Abbreviations: I: Intermediate; R: Resistant; S: Susceptible; MIC: Minimum inhibitory concentration

According to the expert statement published in 2020, early adequate antimicrobial therapy is a key to improve patient outcomes, especially in those with criteria for sepsis or septic shock [16]. Carbapenems were the best choice based on the local surveillance data of antimicrobial resistance, in that critical condition. In the present case, the patient was treated with imipenem during the initial stage of fever, following which the leukocyte count decreased slightly. As the inhibition zone diameter of imipenem(10 µg) was 6 mm, tested by Kirby-Bauer method, the rapid antimicrobial susceptibility test result of blood culture showed resistance to it. Such situation indicated that the sepsis might induced by polymicrobial infection. Procalcitonin decreased rapidly from 100 ng/mL to 34.78 ng/mL, and the body temperature reduced from 40℃ to 38℃, which proof that the anti-infection therapy were effective. Because the patient was in a critical condition and had respiratory failure, imipenem administration was continued to control the infection empirically. On the 4th day after the operation, cefoperazone-sulbactam was administered instead of imipenem on the basis of the antimicrobial susceptibility test results of *R. pickettii*. The rapid antimicrobial susceptibility test results for imipenem were quite different from the final results, which attracted the attention of our microbiologists. The Vitek 2 system and Phoenix M50 were used to determine antimicrobial susceptibility, and the obtained results were consistent. However, even after the administration of antibiotics, the situation became worse since the 5th day after the operation. At this time, in addition to *R. pickettii*, a minute colony was found in the culture plate of the first two positive culture bottles, which was consistent with the colonies obtained from the other three positive blood cultures in succession. Because of the slow growth, the colony was detected 2 days after the inoculation of the sample. The strain initially grew as a tiny white colony, which gradually became yellow after 48 h. The analysis of 16SrRNA sequences revealed 99.7% similarity between the isolate and *M. laevaniformans.* The *Microbacterium* was found in lesser amount in the positive blood culture, which took longer time to generation comparing to *Ralstonia*, in order to be observed on Gram stain. The difference in number of them prevented us to notice two types of bacteria pathogens in the blood smear promptly. The MIC values of *Microbacterium* was determined by the Etest (based on the CLSI m45 *Corynebacterium* breakpoint) as follows: penicillin, 0.25 µg/mL (intermediary); levofloxacin, 0.19 µg/mL (no breakpoint); vancomycin, 0.38 µg/mL (sensitive); linezolid, 0.75 µg/mL (sensitive); meropenem > 32 µg/mL (resistant).

Kathrina Gneiding et al. isolated *Microbacterium* from 50 clinical samples in 5 years and found that all 50 strains were sensitive to linezolid and meropenem. Only one strain was resistant to vancomycin, and one strain was resistant to doxycycline. Ciprofloxacin showed the weakest antimicrobial activity against these strains. A total of 22% of the strains were moderately sensitive, while 22% strains were drug-resistant [9]. According to a previous study on 69 *Microbacterium* strains, the resistance rate to vancomycin and meropenem was 26% and 71%, respectively [7], while the strain isolated in the present case was resistant to meropenem.

*Microbacterium* can grow under anaerobic conditions, while *R. pickettii* is a strictly aerobic species. Therefore, of the 4 blood culture bottles collected 2 h after the operation, 2 culture bottles yielded positive results; both these bottles were cultured aerobically, and the growth of two different bacterial species was observed. The other 3 bottles collected 2 days after surgery yielded positive results in succession; these included 2 bottles cultured aerobically and 1 bottle cultured anaerobically, in which *Microbacterium* alone was cultured. Because imipenem was administered in the early stage of infection, *R. pickettii* was not cultured in subsequent bottles that tested positive. In the later stage, the patient was treated with a combination of levofloxacin and cefoperazone-sulbactam, and her health condition improved significantly; the blood culture also yielded a negative result. After 15 days, she was transferred from ICU to the general ward and discharged after 54 days of rehabilitation training.

Few studies have reported epidemiological data and clinical characteristics of infections caused by different species of *Microbacterium*. The infection route and pathogenetic mechanism in clinical patients are, however, not clear. Some studies believe that *Microbacterium* can cause catheter-related infection, internal fixation device infection [17, 18] or endocarditis [19]. It can also cause nosocomial infection outbreaks [20] and form biofilms similar to *Ralstonia*. Chlorine-containing disinfectants at certain concentrations can inhibit the growth of *M. laevaniformans*. The present case is the first case of bloodstream infection caused by *R. pickettii* and *Microbacterium* together.

Because of the slow growth of *Microbacterium*, its detection can be easily missed in the early stage of treatment and was hard to purify. Following the identification of *R. pickettii*, it was found that the drug sensitivity results of carbapenems were quite different from the rapid antimicrobial susceptibility test results of blood culture; moreover, the patient’s infection worsened following a short improvement, which indicated that the infection was not effectively controlled. This finding confirmed the presence of two different types of bacteria pathogens in blood culture. In clinical treatment, antibiotics are often used empirically after the blood culture shows positive results. The de-escalation therapy should be guided by the antimicrobial susceptibility test results.The rapid antimicrobial susceptibility test results of blood culture have therefore an important reference value in treating patients with such bloodstream infections.The results have been confirmed correlate well with those of standardized test in studies [21]. Difference between the two tests could make us pay attention on it, which could be treated as another potential benefit. Bacteria slow-growing are more likely to be detected after the susceptibility test results of growth advantage one being reported, when the polymicrobia infection occurred. The result of rapid antimicrobial susceptibility tests could be an important reference for clinicians to adjust the antibiotic management.

Numerous reports in the medical literature have highlighted the presence of *Ralstonia pickettii* and *Microbacterium* infection in patients of underlying immune deficiencies, with for example those with hematological malignancies, patients in intensive care, and frail older adult etc. [4, 7, 11, 22]. Patient in our investigation was female, 55 years old with Hashimoto’s thyroiditis poorly controlled. Hahimoto’s thyroiditis (HT)is the chronic inflammation of the thyroiditis which also named chronic lymphocytic thy-roiditis, as the most common autoimmune disease worldwide. Healthy gut mirobiota might be important to gastrointestinal homeostasis and metabolism, and involved in a rage of diseases, including autoimmunity [23]. Thyroid-gut axis has been proposed, as a lot of evidence that the HT development were promoted by the intestinal dysbiosis, bacterial overgrowth, and increased intestinal permeability. The patient with HT exist leaky gut [24]. Whether research on relationship between gut mirobiota and autoimmune disease could be treated as a basis for the source of other opportunistic pathogenic bacteria such as *Ralstonia* and *Microbacterium*?

According to the degree of postoperative fever and the rate of onset of the patient, it is considered that a large number of bacteria quickly enter the blood, causing the patient to quickly enter the state of septic shock. No pathogen was isolated in the urine, surgical incision and sputum samples collected from the patient. The infection event of the patient is the most likely during the operation. Indeed, there have been reports that *Ralstonia* bacteria can survive in liquid media and in hospital settings and devices [25], as an opportunistic pathogen. Since the intraoperative pathology was not tested for bacterial culture, the operating room environment and instrument culture were negative. Although variety specimens covered all potential sources related collected for surveillance cultures were free from both *R. pickettii* and *Microbacterium* during several days. The source of infection could not be determined. Regardless of the source of the infection, the critical sepsis shock was due to them. If the patient suffered sepsis without identified origin, a variety of samples specimens should be collected to culture including the tissue removed in surgical even if the clinician considered it sterile.

In the end, we suggest paying attention to the results of rapid antimicrobial susceptibility test and standardized test. Those may indicate some important situations. Laboratory technicians need to carefully analyze unusual results encountered in the work, like a detective, and communicate with clinician in time. The main limitation of this study is the results of *Microbacterium* were extrapolating MIC interpretative criteria from conforms which lack of sufficient support. It is unknown whether our patient’s *R. pickettii* and *Microbacterium* have acquired or intrinsic resistance to meropenem. Further studies required to verify the mechanism of resistance. Researches of investigating and characterizing the gut microbial composition of HT patients with opportunistic pathogenic bacteremia could be treated as a basis for clarifying the potential infection source.

## Data Availability

The datasets generated and analyzed during the current study are available in the NCBI GenBank repository, under accession number PP439501.1(https://www.ncbi.nlm.nih.gov/nuccore/PP439501).
